# Exploring rigid-backbone protein docking in biologics discovery: a test using the DARPin scaffold

**DOI:** 10.3389/fmolb.2023.1253689

**Published:** 2023-08-24

**Authors:** Francis Gaudreault, Jason Baardsnes, Yuliya Martynova, Aurore Dachon, Hervé Hogues, Christopher R. Corbeil, Enrico O. Purisima, Mélanie Arbour, Traian Sulea

**Affiliations:** ^1^ Human Health Therapeutics Research Centre, National Research Council Canada, Montreal, QC, Canada; ^2^ Institute of Parasitology, McGill University, Montreal, QC, Canada

**Keywords:** binding affinity, protein-protein docking, rigid backbone, DARPin, ProPOSE

## Abstract

Accurate protein-protein docking remains challenging, especially for artificial biologics not coevolved naturally against their protein targets, like antibodies and other engineered scaffolds. We previously developed ProPOSE, an exhaustive docker with full atomistic details, which delivers cutting-edge performance by allowing side-chain rearrangements upon docking. However, extensive protein backbone flexibility limits its practical applicability as indicated by unbound docking tests. To explore the usefulness of ProPOSE on systems with limited backbone flexibility, here we tested the engineered scaffold DARPin, which is characterized by its relatively rigid protein backbone. A prospective screening campaign was undertaken, in which sequence-diversified DARPins were docked and ranked against a directed epitope on the target protein BCL-W. In this proof-of-concept study, only a relatively small set of 2,213 diverse DARPin interfaces were selected for docking from the huge theoretical library from mutating 18 amino-acid positions. A computational selection protocol was then applied for enrichment of binders based on normalized computed binding scores and frequency of binding modes against the predefined epitope. The top-ranked 18 designed DARPin interfaces were selected for experimental validation. Three designs exhibited binding affinities to BCL-W in the nanomolar range comparable to control interfaces adopted from known DARPin binders. This result is encouraging for future screening and engineering campaigns of DARPins and possibly other similarly rigid scaffolds against targeted protein epitopes. Method limitations are discussed and directions for future refinements are proposed.

## 1 Introduction

Biologics have witnessed a tremendous growth in the past decades, with antibody-based therapeutics leading the way and recombinant proteins forming another important market segment (DeFrancesco, 2019; Lu et al., 2020; Kaplon et al., 2023). Advances in computational methods have spurred the idea that in the not-so-distant future, novel biologics can be discovered entirely *in silico*, complementing current wet-lab methods such as immunization and display technologies. This emerging field is dubbed *de novo* discovery of biologics with a particular emphasis on *de novo* antibody engineering (Fischman and Ofran, 2018).

Central to this *de novo* discovery approach is the ability to dock and score large libraries of biologic variants on the three-dimensional (3D) structure of a target protein (e.g., the antigen in the case of antibodies). Artificial intelligence/machine learning (AI/ML)-based methods like AlphaFold2 (Jumper et al., 2021), which have recently demonstrated a tremendous success in predicting protein structures and complexes of biologically co-evolved proteins, unfortunately are not applicable to docking and scoring of antibodies and artificially designed proteins (Yin et al., 2022). This limitation is due to co-evolution data being essential to AI/ML’s success in protein-protein docking (Evans et al., 2022; Gao et al., 2022). Compounding the docking and scoring challenge is the difficulty to predict 3D structures of antibody libraries. While there has been some recent success in modeling antibodies with AI/ML methods without co-evolutionary information, there are still challenges in predicting the conformation of the hypervariable CHR-H3 loop (Abanades et al., 2022; Cohen et al., 2022; Ruffolo et al., 2022). Due to technical limitations from the high dimensionality of the CDR-H3 conformational space, the applicability of *de novo* antibody discovery efforts based on docking modeled antibody libraries to an antigen structure was met with limited success, as reported with several classical approaches (Adolf-Bryfogle et al., 2018; Chowdhury et al., 2018; Warszawski et al., 2019; Wood, 2021). Instead, applications on biologics displaying limited amounts of flexibility should be explored for increased likelihood of success (Youn et al., 2017; Radom et al., 2019).

We previously developed ProPOSE, an exhaustive direct protein-protein docker with full atomistic details (Hogues et al., 2018). By allowing side-chain rearrangements upon docking, ProPOSE delivers the current leading-edge performance in both general protein-protein docking and the specific case of antibody-antigen docking, when the backbone conformations of the interacting partners in the complex are *a priori* known. More specifically, ProPOSE maintains a strong performance even when side-chain flexibility is of concern. However, the docking accuracy was lower when backbone atoms experienced significant displacements between the bound and unbound states. We anticipated that despite its limitations, ProPOSE should be able to show utility in *de novo* biologics discovery when there is limited backbone flexibility upon binding and when reasonable models of backbone conformations can be inferred for the library of potential binders.

Hence, in this proof-of-concept study, we turned away from antibodies and towards the well-known engineered scaffold called DARPin (Designed Ankyrin Repeat Protein) (Binz et al., 2003). The DARPin scaffold has been refined over the years and has proven its value for the discovery of molecules with various medical and engineering applications, for example, as biotherapeutics, diagnostic agents, biosensors, molecular probes and crystallization helpers (Pluckthun, 2015; Rothenberger et al., 2022; Strittmatter et al., 2022). Compared to antibodies, DARPins are generally considered to be more rigid due to their smaller size and more defined structure. The repeating ankyrin unit (a β-turn followed by two anti-parallel α-helices) confers rigidity and stability to their structure (Kramer et al., 2010; Schilling et al., 2022). Such a limited backbone flexibility thus appears suitable for modeling DARPin substitution variants relatively reliably starting from available DARPin template structures.

Hence, the exploratory prospective study described here was centered around applying ProPOSE rigid-backbone docking to the DARPin scaffold exhibiting relative backbone rigidity. A computational flow was devised to generate a relatively small library of diverse DARPin interfaces for directed docking to a known epitope on the structure of the protein target, BCL-W. A selection procedure was further devised to establish a score threshold that captured self-consistent positive controls generated within the same computational procedure. Prospective computational designs were then subjected to experimental testing. Testing of 18 top-ranked hits demonstrated that half of them had detected binding to the target. Comparative analysis of computational and experimental data prompted to several limitations and areas for future improvements of the rigid-docking based approach for *de novo* biologics discovery.

## 2 Materials and methods

### 2.1 Computational methods

**FIGURE 1:**
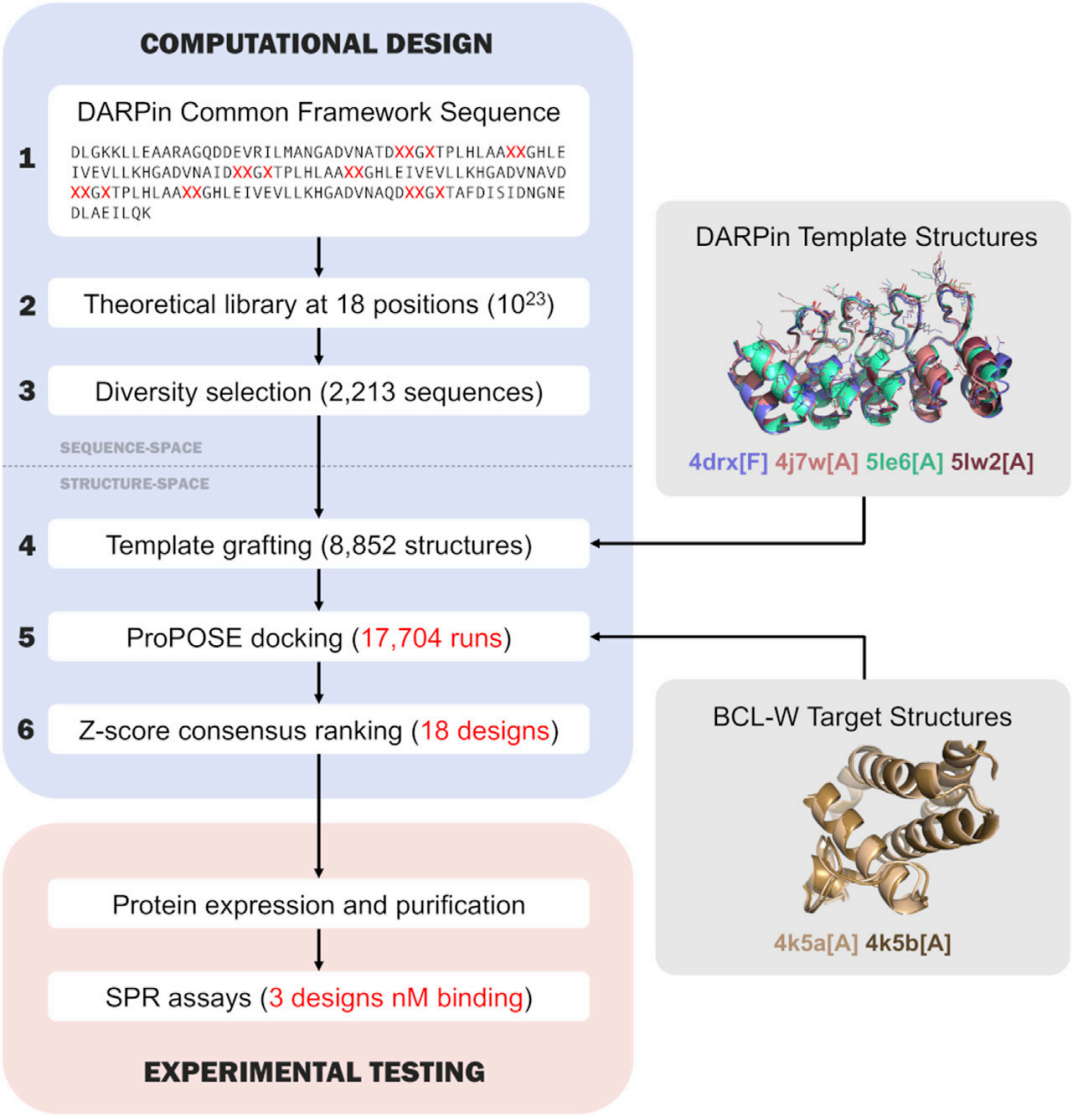
Flowchart of the overall computational design and experimental testing. The first three steps of the computational design are in the sequence space, while the last three steps are in the 3D-structure space and inherit structural knowledge from the Protein Data Bank (Berman et al., 2000). The main steps of the computational design are numbered outside the boxes and described in the text.

The sequence-based and structure-based computational design process (Figure 1) consisted of 6 steps which are described in the following sub-sections.

#### 2.1.1 Defining the DARPin common framework sequence

Hundreds of DARPin structures with various topologies were published in the literature and are accessible in the PDB, among which many have 4 or 5 repeated ankyrin motifs. Two DARPins evolved through ribosome display to bind BCL-W, and corresponding to PDB entries 4k5a and 4k5b (Schilling et al., 2014b), were used as known binders in this study. These known binders engage the target in a binding mode which is typical for DARPins, which consists of interactions made by the concave paratope formed by their 5 repeated ankyrin motifs (Binz et al., 2003; Kramer et al., 2010; Pluckthun, 2015; Schilling et al., 2022). By inspecting the sequences and structures of these known binders and other DARPins with available crystal structures in PDB, a common framework sequence was defined for further library expansion. The main features considered during the selection of a DARPin common framework sequence were: 1) 157 amino acids starting with DLGKK and ending with LQKAA sequences; 2) conserved regions at these N- and C-terminal ends; 3) consensus residues deemed essential for the stability of the overall fold along repeated ankyrin motifs; and 4) key residues contributing to binding along repeated ankyrin motifs. These criteria led to a single DARPin common framework sequence, which corresponded to the DARPin of chain F in the PDB entry 4drx (the nomenclature 4drx [F] is used) (Pecqueur et al., 2012).

#### 2.1.2 Expanding the framework sequence into a DARPin library

**FIGURE 2:**
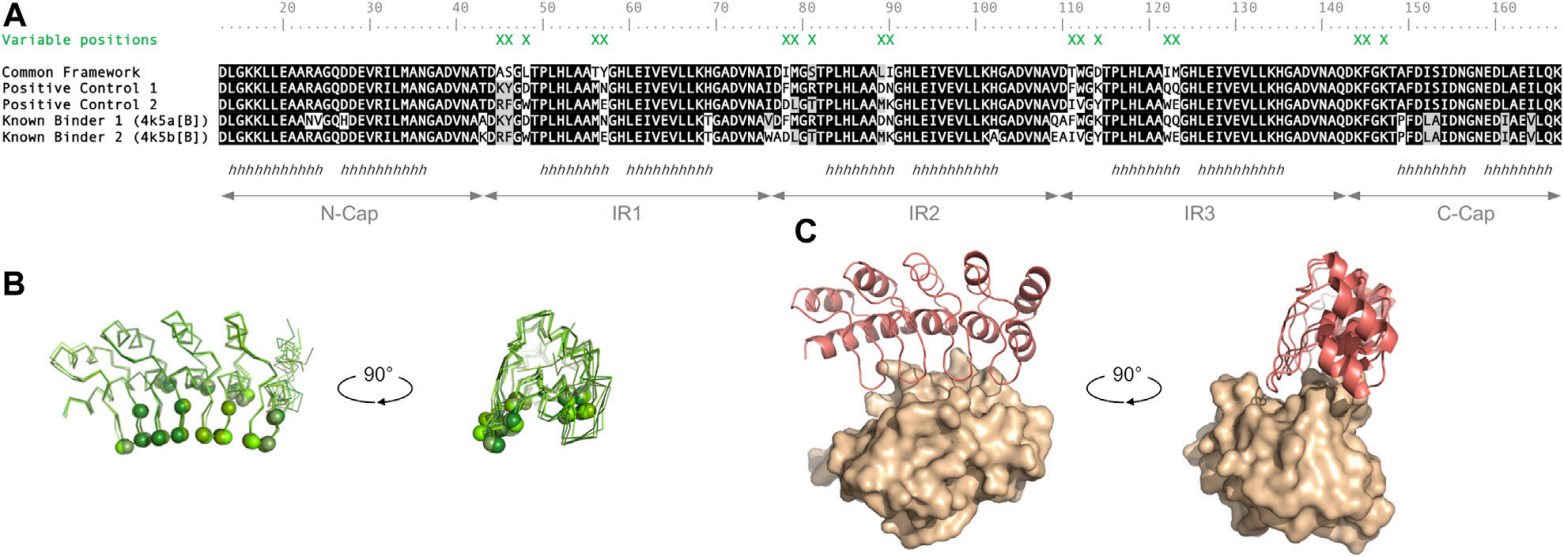
Variable positions on the DARPin scaffold docked onto BCL-W target epitope. **(A)** Sequence alignment between the common framework sequence (4drx [F]), known binders (4k5a [B] and 4k5b [B]), and the positive controls (PC1 and PC2) grafting the interface of the known binders onto the common template sequence, at the 18 variable positions (marked by green Xs). The conventional DARPin sequence numbering scheme is used, *h* denotes α-helix, IR1 to IR3 delineate internal ankyrin repeats 1-3, and N-Cap and C-Cap are the terminal ankyrin repeats. **(B)** Location of the 18 variable positions (spheres) on the 4 DARPin template structures (Cα-traces with different shades of green). **(C)** Location of the docking site on the BCL-W target protein indicated by the crystal structure (4k5b) of a known DARPin binder (red cartoon) complexed with the BCL-W target (molecular surface).

A set of 18 amino-acid positions within the defined DARPin common framework sequence were manually selected and allowed to vary (referred to as variable positions). These positions, which have high-frequency rates of mutation as observed from sequence alignments of many DARPins from the literature, are: 45, 46, 48, 56, 57, 78, 79, 81, 89, 90, 111, 112, 114, 122, 123, 144, 145 and 147 (standard DARPin numbering is applied). Amino-acid side chains at these positions are lining the concave face of the DARPin scaffold by being located within the β-turn loops and following short α-helices of the ankyrin repeats (Figure 2).

Two positive controls having the 18 variable positions corresponding to known DARPin binders of BCL-W, having PDB entries 4k5a [B] and 4k5b [B], were also built manually into the library. It is important to note that these constructed positive controls share the common framework of the designed library described above and thus differ at several positions from the frameworks of the originating known binders (Figure 2).

#### 2.1.3 Selecting a DARPin sub-library of diverse sequences

An alphabet was created to group amino acids by chemical properties. The following five groups excluding Gly, Cys and Pro were defined: positively-charged (Arg, His, Lys); negatively-charged (Asp, Glu); polar (Asn, Gln, Ser, Thr); non-polar (Ala, Ile, Leu, Met, Val); and aromatic (Phe, Trp, Tyr). Equal probability was given to each group to be selected when mutating sequences. Similarly, amino acids within a group were given equal probability.

The designs were generated using a stochastic procedure in which variable amino-acid positions were mutated either through point mutations or through permutations of amino acids. Multiple starting points in the sequence space were used to generate the designs. The set of mutated designs (M-set) were generated starting from the 4drx [F] sequence chosen as common framework. All variable amino acids were forced to be mutated in this set. To be included in the library, a design sequence had to be sufficiently distant to the designs comprised within the same set. A threshold distance of 10 was fixed which required at least 10 alphabet group changes. The set of permutated designs (P-set) were generated starting from the sequences of the two positive controls. No change in the alphabet group was imposed for this set. A threshold distance of 13 was set, requiring at least 13 amino-acid changes.

#### 2.1.4 Grafting DARPin sequences onto template structures

The designed sub-library sequences were grafted onto four DARPin template structures followed by side-chain repacking using SCWRL4 (Krivov et al., 2009). The last two alanine residues at the C-terminus of the template sequence were truncated for modeling purposes. Only those side-chains that are different at a given side-chain were mutated and repacked to preserve the structural integrity of the original crystal structures of the DARPin templates. The entire structure was then allowed to be repacked. The DARPin templates from the following PDB entries were used in this study: 4drx [F], 4j7w [A], 5lw2 [A] and 5le6 [A] (Figure 1). The backbone structures of these templates are distinct from those of the two known DARPin binders of BCL-W, 4k5a [B] and 4k5b [B], which were purposely excluded as structural templates to avoid the cognate-docking bias. The selected DARPin template structures underwent the following preparation procedure: 1) addition of missing side chain atoms (no repacking); 2) addition of missing hydrogen atoms and assignment of standard protonation states at pH 7; 3) optimization of the hydrogen-bond network the minH program (Hogues et al., 2014); and 4) AMBER force-field (Cornell et al., 1995; Hornak et al., 2006) energy minimization of added hydrogen atoms and any newly added side-chain atoms with harmonic restraints on all the other heavy atoms of 1,000 kcal/mol/ ${\overset{{}^{\circ}}{A}}^{2}$ followed by energy minimization of the entire structure with harmonic restraints of 10 kcal/mol/ ${\overset{{}^{\circ}}{A}}^{2}$ on backbone heavy atoms, 1 kcal/mol/ ${\overset{{}^{\circ}}{A}}^{2}$ on side-chain heavy atoms, and no restraints on hydrogen atoms.

#### 2.1.5 DARPin docking protocols

The BCL-W docking-based screening of the DARPin library was performed using the exhaustive docking engine ProPOSE version 1.03 (Hogues et al., 2018). ProPOSE was run with default parameters using the HITSET flag to force binding towards the set of residues involved in binding BCL-W. Initially, no binding location (or epitope) was defined on the target protein BCL-W and exhaustive docking was performed all around the BCL-W structure. Two BCL-W structures were employed for docking, with PDB entries 4k5a [A] and 4k5b [C] (Figure 1), which correspond to the BCL-W complexed with the two known DARPin binders. For each DARPin library sequence, the four DARPin structural templates carrying the grafted designed sequence were docked against the two BCL-W target structure, resulting in 8 docking experiments. In this study, only the top-1 scored pose generated by ProPOSE was considered for a given complex given its accuracy in pose recovery as top-1 when the protein backbone conformation is known, without the need for rescoring (Hogues et al., 2018). On average, a single docking run took 30 min to execute when parallelized on an Intel Xeon Gold 5,218 using 6 cores.

Epitope restriction on the BCL-W target was introduced after all docking calculations were completed. In this proof-of-concept study, we elected to target the same BCL-W epitope and the DARPin binding mode observed for the two known BCL-W DARPin binders (PDB entries 4k5a and 4k5b) (Schilling et al., 2014b). The similarity of predicted docked poses of designed sequences relative to these known structures was based on CAPRI classification (Lensink et al., 2017). Predictions were compared on the basis of: 1) the backbone RMSD of the ligand upon target superposition; 2) the backbone RMSD of the interface upon superposition of interface atoms; and 3) the fraction of preserved contacts (f_con_). The ligand and target were DARPin and BCL-W, respectively. Noteworthy, f_con_ was used rather than the standard f_nat_ from CAPRI that is derived from the comparison to a native structure. Moreover, f_con_ is a position-dependent (amino acid-independent) measure allowing designs with different sequences to be compared. Two predictions were declared as having high, medium or acceptable quality, or as incorrect otherwise, with thresholds defined by the CAPRI classification (Lensink et al., 2017).

#### 2.1.6 Ranking docked DARPin structures

The number of top-1 scored poses, N_pose_, docked at the targeted epitope from the 8 docking runs was used to retain only those designs that have at least 2 poses docked at the target epitope. To this end, the predicted poses for a given DARPin were grouped using a greedy clustering algorithm with a tolerance of at least medium quality between cluster representatives. In geometric terms, for poses to be considered bound at the targeted epitope occupied by one of the known binders, they were required to have acceptable quality criteria, i.e., 1) f_con_ of at least 30% with a ligand backbone RMSD >5.0Å and interface backbone RMSD >2.0 Å; or alternatively, 2) f_con_ between 10% and 30% while having a ligand backbone RMSD <10.0 Å or an interface backbone RMSD <4.0 Å. For poses to be part of the same cluster, they were required to have medium quality criteria, i.e., 1) f_con_ of at least 50% with ligand backbone RMSD >1.0 Å and interface backbone RMSD >1.0 Å; or alternatively, 2) f_con_ between 30% and 50% while having ligand backbone RMSD <5.0 Å or an interface backbone RMSD <2.0 Å. No cut-off in score was applied for the clustering.

For each design with N_pose_ > 1, a consensus score was derived as the arithmetic average over the docking scores of the poses binding to the targeted epitope. Consensus scores over the designs with N_pose_ > 1 were also normalized into Z-scores to better inform the selection of a top-ranked population based on a minimum number of standard deviations away from the mean calculated from the distribution of all DARPins combining the P-set designs, M-set designs and the positive controls.

#### 2.1.7 Other software and data availability

Structure visualization was performed in PyMOL (The PyMOL Molecular Graphics System, Version 2.0, Schrödinger, LLC). Statistical analyzes were run in R (R Development Core Team, 2011). ClustalW2 was used to run the multiple sequence alignments (Larkin et al., 2007).

The sequence datasets generated for this study have been made available as a MongoDB with example scripts that can be found at the GitHub repository https://github.com/gaudreaultfnrc/Darpins.

### 2.2 Experimental methods

#### 2.2.1 Protein expression and purification

Each DARPin design included a N-terminus tag (MRGSHHHHHHGS) and two alanines at their C-terminus as described in (Schilling et al., 2014a). The protein sequences were optimized for *Escherichia coli* expression using a multifactor algorithm (https://www.genscript.com/tools/gensmart-codon-optimization), then synthesized by GenScript. After inserting each gene in pET24a (+) via NdeI and NotI restriction enzyme sites, the final plasmids were transformed into NRC *E. coli* BL21-T7 strain (*rhaB lacZ*::P*tac*-T7 RNAP). For each clone, a 2.8-L Fernbach baffled flask containing 500 mL Animal-Product Free (APF) LB Miller (Athena Enzyme Systems Cat. 0133) plus 50 μg/mL kanamycin was inoculated with an overnight preculture to get an initial OD_600nm_ of 0.1. The flasks were incubated at 37°C, 200–250 rpm until an OD_600nm_ between 0.8 and 1.0 were reached. To induce protein expression 1 mM isopropyl β-d-1-thiogalactopyranoside (IPTG) was added and the culture incubated for another 4 h at 37°C, 200–250 rpm. The cultures were harvested, and the cell pellets stored at −80°C.

Before purification, a cell pellet was resuspended in Lysis buffer 50 mM NaPO_4_, 300 mM NaCl, 10 mM imidazole, pH 7.4 with cOmplete protease inhibitors EDTA-free (Millipore Sigma Cat. 11836170001) and lysed by two passages on a French Pressure Cell Disruptor. Finally, the cell lysate was clarified by centrifugation at 10,000 x *g*, 4°C, for 15 min and filtration on 0.45 µm filter. A fraction of the clarified lysate (15 mL) was applied on a 3 mL HisPur Cobalt Spin Column (Thermo Fisher Cat. 89969) and the column was washed with 20 mM NaPO_4_, pH 7.5, 500 mM NaCl, 0.3 mM TCEP, 15 mM imidazole. Elution was done with 20 mM NaPO_4_, pH 7.5, 500 mM NaCl, 0.3 mM TCEP, 100 mM imidazole and pooled after visualization on SDS-PAGE. For some of the proteins, the purification was repeated to increase purity. Buffer exchange for DPBS (Thermo Fisher Cat. 14190144) was done with PD-10 desalting columns (Cytiva Cat. 17085101) and final concentration measured by Qubit Protein Assay (Thermo Fisher Cat. Q33211).

The design of BCL-W was based on (Schilling et al., 2014a) with an N-terminal Avi-tag followed by a bacteriophage lambda protein D fusion tag to improve protein solubility (Forrer and Jaussi, 1998) (see Supplementary Data). A 6xHis tag was added to the C-terminus of BCL-W for purification. Gene optimization, synthesis and cloning in pET24a (+) vector was done as described above for the DARPins. To allow *in vitro* biotinylation, the NRC *E. coli* BL21-T7 strain (*rhaB lacZ*::P*tac*-T7 RNAP) was first transformed with pBirAcm (Avidity), a plasmid expressing biotin ligase under *tac* promoter (IPTG inducible). After growing a chloramphenicol resistant colony in APF LP Miller medium containing 10 μg/mL chloramphenicol, electrocompetent cells were prepared using standard procedures. The plasmid pET24a (+)-BCL-W was then transformed in BL21-T7/pBirAcm strain and selected on APF LB Miller agar containing 50 μg/mL kanamycin and 10 μg/mL chloramphenicol.

Expression of BCL-W, cell lysis and clarification were done as described for the DARPins with some exceptions. Both antibiotics, kanamycin and chloramphenicol, were used, and biotin was added to a final concentration of 5 mM during the culture (25 mL). The cells were lysed in a buffer containing 50 mM NaPO_4_, 300 mM NaCl, 10 mM imidazole, pH 8.0 (plus cOmplete EDTA-free protease inhibitors). The clarified lysate (2.5 mL) was applied on a 0.2 mL HisPur Cobalt Spin Column (Thermo Fisher Cat. 90090) and the column was washed with 20 mM NaPO_4_, pH 7.5, 500 mM NaCl, 0.3 mM TCEP, 20 mM imidazole. Elution was done with 20 mM NaPO_4_, pH 7.5, 500 mM NaCl, 0.3 mM TCEP, 300 mM imidazole and pooled after visualization on SDS-PAGE. Buffer exchange for DPBS (Thermo Fisher Cat. 14190144) was done with G-25 MiniTrap desalting columns (Cytiva Cat. 28918007) and final concentration measured by Qubit Protein Assay (Thermo Fisher Cat. Q33211). Purity levels are given in Supplementary Table S1 and SDS-PAGE gels are provided as Supplementary Data.

#### 2.2.2 Binding affinity measurements

Surface plasmon resonance was used to screen the top 18 DARPin designs for binding to the biotinylated BCL-W using a Biacore T200 instrument (Cytiva Inc., Marlborough MA) at 25°C and with PBST running buffer (Teknova, Hollister CA) containing 0.05% Tween 20, 3.4 mM EDTA and an additional 350 mM NaCl. The strategy employed was to capture the biotinylated BCL-W onto the SPR surface with a CAP sensor chip (Cytiva Inc.) and flow a three-point concentration series of the DARPin scaffold using a 10-fold dilution series from 1 μM to cover a wide concentration range. From the resulting sensorgrams, the affinity constant of binding candidates can be determined. A CAP immobilization chip was prepared following the manufacturer’s instructions. Each injection cycle consisted first of a 120-s injection at 5 μL/min of a 5-fold dilution of CAP reagent to indirectly immobilize streptavidin over flow-cells 1 and 2. This was followed by a 240-s capture of 5 μg/mL biotinylated BCL-W at 5 μL/min over flow cell 2 only to form the 60–62 RU BCL-W surface, and finally a three-point concentration injection of the DARPin scaffold or running buffer only using single-cycle kinetics was performed at 50 μL/min for 90 s with a 300-s dissociation phase. At the end of the dissociation phase, any BCL-W/DARPin complex was stripped from the SPR surface using a 60-s injection of 6 M GuCl/0.25 M NaOH taken from the CAP sensor chip reagent kit. The sensorgrams were double referenced and analyzed using the Biacore BiaEval software. Affinities of the DARPin scaffolds for BCL-W were determined using the steady state model, or the 1:1 binding model when kinetic rate constants could be evaluated.

#### 2.2.3 Folding stability measurements

Differential scanning calorimetry (DSC) was used to determine the thermal transition midpoints (T_m_) as previously performed (Schrag et al., 2019). DSC was carried out in a VP-Capillary DSC system instrument (Malvern Instruments Ltd., Malvern, United Kingdom). Samples were diluted in DPBS buffer to a final concentration of 0.4 mg/mL. DPBS blank and sample scans were carried out by increasing the temperature from 20°C to 100°C at a rate of 60°C/h, with feedback mode/gain set at “low”, filtering period of 8 s, pre-scan time of 3 min, and under 70 psi of nitrogen pressure. All data were analyzed with Origin 7.0 software (OriginLab Corporation, Northampton, MA). Thermograms were corrected by subtraction of corresponding DPBS blank scans and normalized to the protein molar concentration. The T_m_ values were determined using automated data processing with the rectangular peak finder algorithm for T_m_. Melting temperatures are listed in Supplementary Table S1 and DSC thermograms are provided as Supplementary Data.

## 3 Results

### 3.1 Sequence-based and structure-based computational design

#### 3.1.1 Overall design process

The flowchart in Figure 1 presents the overall computational design process devised and implemented for this rigid-docking based proof-of-concept engineering study based on the DARPin scaffold. It includes 6 steps: 1) definition of a single DARPin common framework sequence; 2) expansion of the common framework sequence into a DARPin sequence library with variable positions; 3) selection of a small DARPin sub-library consisting of diverse sequences; 4) grafting of the sequence sub-library onto DARPin structural templates; 5) docking of DARPin sub-library to target protein structures, the core component of the process; and 6) ranking docked DARPin variants for experimental testing. The first three steps operate in the sequence space, whereas the last three in the 3D structure space. All the steps are described in detail in the sub-sections of the Methods section. The following sub-sections focus more in-depth on results obtained in steps 3), 4), 5) and 6) of the process.

#### 3.1.2 Selecting diverse DARPin sub-library sequences

Expanding a common framework sequence by varying 18 positions lining the concave face of the DARPin fold (Figure 2) resulted in 10^23^ theoretical library size. Millions of iterations were run to select a diverse sub-library fulfilling several design criteria (see Methods sub-Section 2.1.3). The resulting diverse sub-library comprised a total of 2,213 designs of which 1,429 were produced by mutations and 784 by permutations (Table 1). The closest designs in sequence are 9 amino-acid substitutions away from any of the two positive controls (Supplementary Figure S1), or 6 groups away when grouping amino acids by homology (see Methods section). The mutation-based designs have an even proportion of amino-acid groups at the variable positions (Supplementary Figure S2). In contrast, permutation-based designs have unevenly distributed amino-acid groups and lack Ala, His and Ser as inherited from the starting positive-control sequences (Supplementary Figure S2). In terms of net charge, mutation-based designs span a wide range from −16 to +3 with a mean net charge of −6.8, whereas permutation-based designs inherit the net charges of their respective parental positive control (Supplementary Figure S3).

**TABLE 1:** Library design statistics.

Starting DARPin	PDB ID	Variable positions^a^	Set^b^	N_seq_ ^c^	d_seq_ ^d^	d_chemseq_ ^e^	Q_net_ ^f^
Common framework	4drx [F]	ASLTYIMSLITWDIMKFK	M	1,429	9	7	−6.8
Known binder	4k5a [B]	KYDMNFMRDNFWKQQKFK	P	284	12	7	−4.0
Known binder	4k5b [B]	RFWMEDLTMKIVYWEKFK	P	500	9	6	−6.0

^a^ Position IDs, in the same order: 45, 46, 48, 56, 57, 78, 79, 81, 89, 90, 111, 112, 114, 122, 123, 144, 145 and 147.

^b^ M: mutation; P: permutation.

^c^ Number of sequences.

^d^ Closest distance from a design to a known binder interface at 18 variable positions, expressed as number of substitutions.

^e^ Closest distance from a design to a known binder interface at 18 variable positions, expressed as number of homology group changes.

^f^ Mean net charge of designs within the set.

**FIGURE 3:**
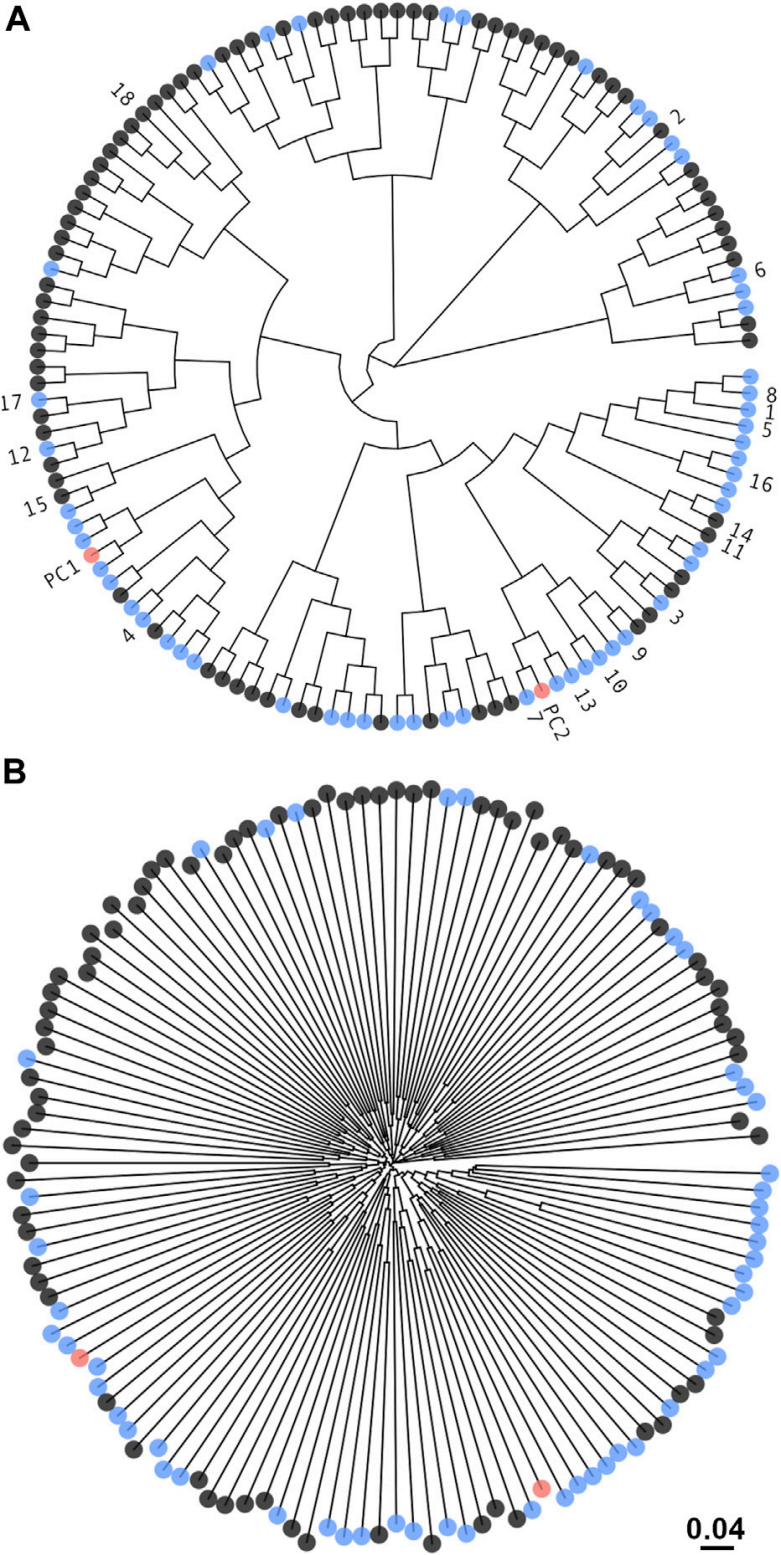
Diversity of the DARPin sequence sub-library. Unrooted phylogenetic tree from hierarchical clustering of sequences by the chemical properties of amino acids using defined amino-acid homology groups (see Methods section). Sequences marked in black are from the mutation-based set and in blue from the permutation-based set. The two positive-control sequences are shown in red. Only a 5% random sample of the sub-library consisting of 134 sequences is plotted. The top-18 consensus designs and positive controls were annotated. **(A)** For visual clarity, the terminal branches were equally trimmed down to a cladogram giving the illusion of sequence proximity (Yu, 2020). **(B)** The non-trimmed tree that preserves the ordering in **(A)** is shown to illustrate the true divergence in sequence between designs. For reference, the evolutionary distance is shown.

In order to generate a sub-library that samples homogeneously the immense theoretical sequence space, designs were imposed to be orthogonal to each other. Clustering based on amino-acid properties indicated that most sequence space regions were covered by both mutation-based and permutation-based types of sequences, with a few areas only covered by the mutation-based set (Figure 3). While proximity in sequence might be perceivable between some of the designs and the two positive controls (Figure 3A), overall, the designed sequences were diverse and nearly equidistant from each other (Figure 3B).

#### 3.1.3 Grafting sequence sub-library onto DARPin structural templates

Four crystal structures were used as templates in the modeling of the DARPin ligands (see Methods section). The variance in RMSD among these templates has a mean of 0.95 Å. The template 4drx [F] is more distant due in part to an opening of the last repeated motif of the scaffold. The magnitudes of backbone changes between each of these templates and any of the 2 known DARPin binders of BCL-W are larger than between the 2 known binders (0.42 Å). Thus, backbone RMSDs of 0.91, 0.75, 0.79 and 0.79Å were calculated to the 4k5a [B] known binder, and of 0.96, 0.75, 0.78 and 0.78Å to the 4k5b [A] known binder, for the template structures 4drx [F], 4j7w [A], 5le6 [A] and 5lw2 [A], respectively. More backbone variations could be observed in the unstructured region of the fourth ankyrin repeat, where the known BCL-W binders had a distinct conformational topology at the tip of this loop region. These variations in the templates relative to known binders were critical for testing the method in real-life application mode in which the bound backbone structure will be unknown *a priori*.

#### 3.1.4 Docking DARPin sub-library structures to target

The entire set of sequence designs in the selected sub-library was grafted onto four template structures, then cross-docked against two target (BCL-W) structures, leading to 8 docking runs per DARPin sequence. The two backbone structures used for the target (4k5a [A] and 4k5b [C]) were relatively close from each other, with an RMSD of 0.77 Å. They also engaged their respective known DARPin binders (4k5a [B] and 4k5b [A]) via a well-preserved binding interface with backbone atoms deviating by an RMSD of 0.60 Å. Hence, in this study, the docked poses for novel DARPins were required to bind around the same epitope that is targeted by these two known DARPin binders of BCL-W. In more technical terms, the predicted poses of designed DARPins were required to have an overlap of at least acceptable quality (according to CAPRI classification (Lensink et al., 2017) to either of these known binders. This was met by 1,033 designs (47% of the sub-library), and are referred to as “locus designs”. (Increasing the stringency and imposing at least a medium quality of pose overlap with the known binders reduced the number of locus designs to 559.) We found no bias towards either of the two target BCL-W structures used for docking, as 811 designs docked to structure 4k5a [A] and 632 designs to structure 4k5b [C]. In terms of the template DARPin structures used for docking, 5lw2 [A] was the least successful template structure with 344 docked designs, followed by 380 designs docked on 5le6 [A], 533 on 4j7w [A] and 579 on 4drx [F]. The net charge distribution of the 1,033 locus designs is slightly different relative the entire docked sub-library of 2,213 designs, as it has sharper peaks at the −6 and −4 net charges (Supplementary Figure S3).

#### 3.1.5 Ranking DARPin virtual hits

First, locus design DARPins were filtered based on the number of top-1 scored poses, N_pose_, that were docked at the targeted epitope from the 8 docking runs for each DARPin. A total of 293 locus designs (13% of the sub-library) had at least 2 poses docked at the target epitope. These were retained for further ranking and were called “consensus designs”. The net charge distribution among the consensus designs had even sharper peaks at the net charges −6 and −4, with the majority of consensus designs at charge −6 (Supplementary Figure S3).

**FIGURE 4:**
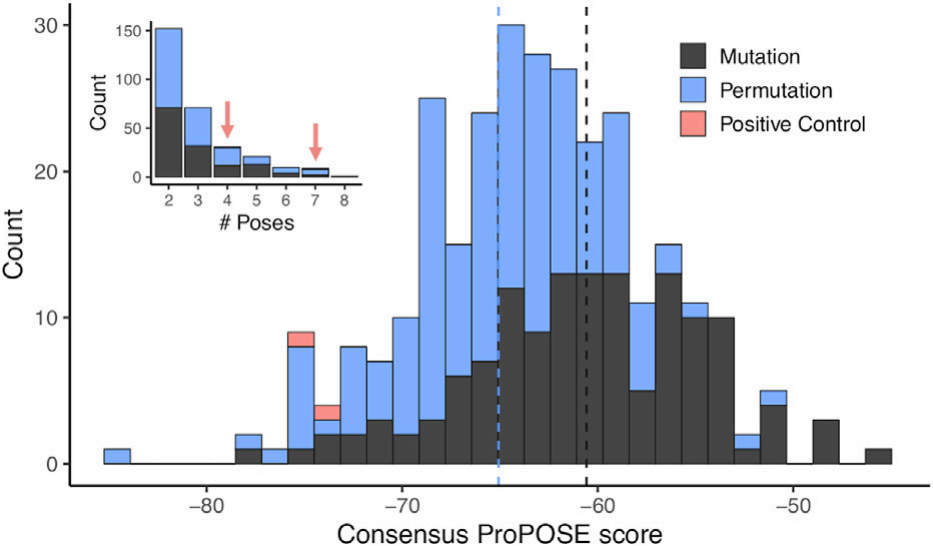
Distribution of scores from docking-based screening. Distribution of scores obtained from the docking experiments using ProPOSE on the entire set of designs in the library. The scores were obtained from a consensus of multiple predictions binding at the same locus while imposing an acceptable or better quality among the representatives of the cluster. The scores follow a normal distribution with the median marked as dashed lines. The underlying area-under-the-curve of the receiver operating characteristic (AUC-ROC) curve obtained from the separation of the two positive controls from the combined mutation and permutation design sets has a value 0.971. The inset shows the distribution in number of representatives used to calculate the consensus ProPOSE score. The two positive controls have 4 and 7 representatives.

**FIGURE 5:**
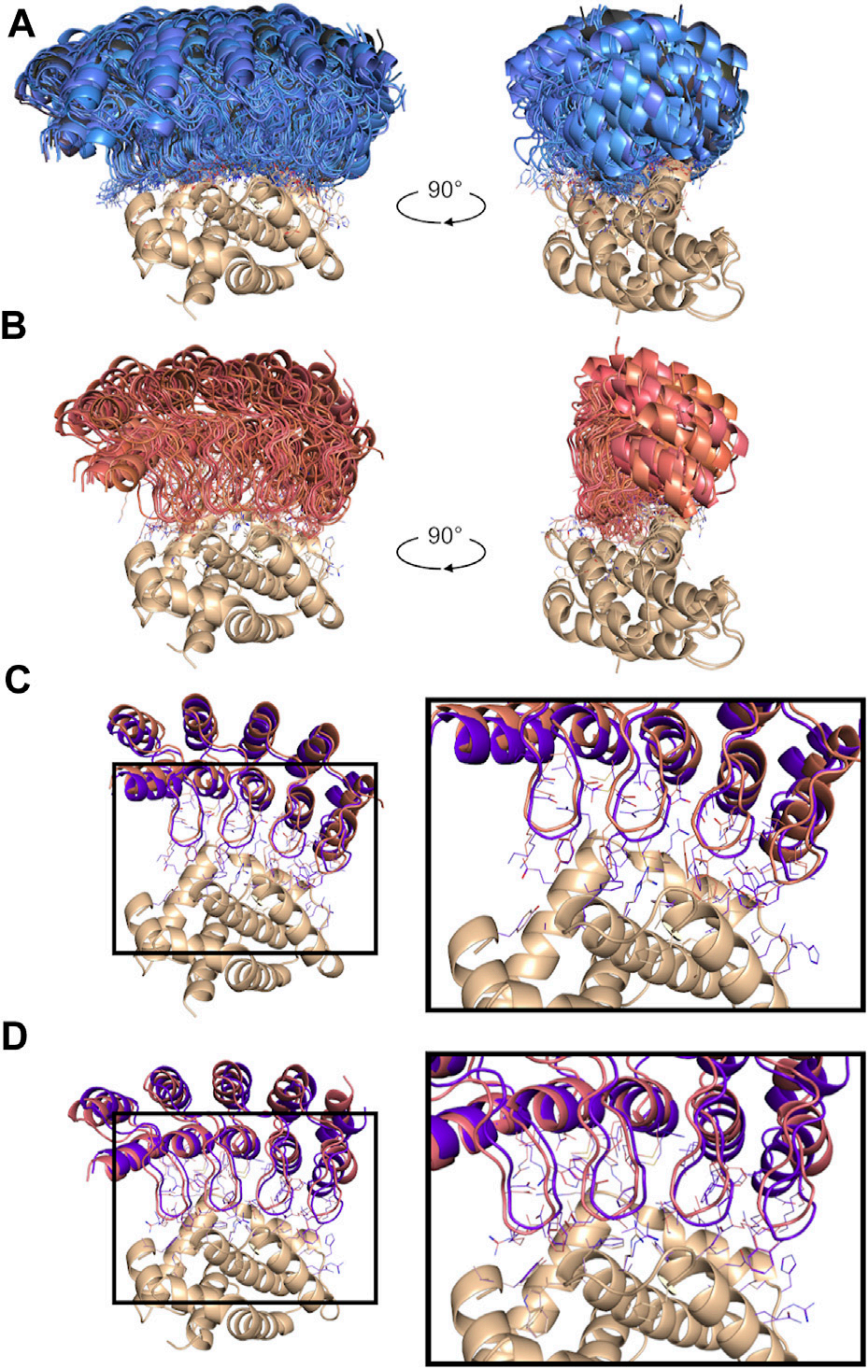
Non-cognate docking results for the top-ranked poses. **(A)** Overview of all poses at the target epitope for the top-18 consensus designs selected for testing. The novel designs are part of the permutation-based set (blue) and the mutation-based set (black). **(B)** Overview of all poses docked at the target epitope for the two positive controls (red). Comparisons of atomic details between the best-scored docked pose of a positive control and the crystal structure of the corresponding known binder sharing the same residues at the 18 variable positions are shown in panel **(C)** for the positive control PC1 and the known binder (4k5a [A]; in purple), and in panel **(D)** for the positive control PC2 and the known binder (4k5b [C]; in purple). All structure orientations are kept as in Figure 2C.

For each of selected 293 consensus designs, a consensus score was derived as the arithmetic average over the docking scores of the poses binding to the targeted epitope. These consensus scores were normally distributed and ranged from −84.2 to −45.3, from strongest to weakest binder (Figure 4). The permutation-based designs were preferentially chosen according to the consensus scores with a median of −65 as opposed to a median of −61 for the mutation-based ones. In total, 152 (52%) and 71 (24%) designs that docked at the targeted epitope did so with values in N_pose_ of 2 and 3, respectively (inset in Figure 4). The lowest consensus score corresponded to a Z-score of −3.5. Consensus designs with Z-scores below −1.5 were selected for experimental validation, which formed a set consisting of 18 novel DARPins (Table 2). An overlay of all consensus poses for the selected designs is shown in Figure 5A.

**TABLE 2:** Top-ranked consensus designs.

Rank	Variable positions^a^	Set^b^	N_sub_ ^c^	N_pose_ ^d^	Q_net_ ^e^	Score^f^	Z-Score^g^	K_D_ (nM)^h^
1	RMTKEKFFWEILWYDMVK	P	14	7	−6	−84.2	−3.5	weak
2	RQIVHRHWFDVIKYWRHL	M	18 (17)	3	−1	−77.8	−2.4	n.d.b
3	KFWFETMDKMKRYEWVIL	P	14	7	−6	−77.2	−2.3	weak
4	KFWMEMLTDWIYEVRKKF	P	10	3	−6	−75.9	−2.1	44
5	KFMREEFWWLIKKTDYMV	P	15	6	−6	−75.3	−2.0	n.d.b
6	KFWYNDFQMDFQMRNKKK	P	13	4	−4	−75.2	−2.0	150
7	VWWEEDFKIKMMKFYTLR	P	14	3	−6	−75.1	−2.0	111
8	KYRKNKFWFNDQFKDQMM	P	14	3	−4	−74.8	−1.9	n.d.b
9	RKMDQKFKMNDYWNFQFK	P	15	2	−4	−74.7	−1.9	weak
10	KIMWFKWDYKELMVETFR	P	15	3	−6	−74.7	−1.9	weak
11	RAVNRTVFVYWAYNFRVV	M	18 (16)	2	−4	−74.6	−1.9	weak
12	KFWMQRFMQYKDFKDKNN	P	15	2	−4	−74.6	−1.9	n.d.b
13	KLMEYDFMVWITKFERWK	P	14	7	−6	−74.6	−1.7	n.d.b
14	KYWYRTTWYHAIWNFYKQ	M	18 (16)	5	−3	−73.5	−1.7	weak
15	KYFEWVQRVMFKVVLMNR	M	18 (14)	2	−4	−73.3	−1.7	n.d.b
16	FKMWEMLFWRVIYEDKKT	P	14	5	−6	−72.8	−1.6	n.d.b
17	KFFRNNKMDYWKKMDFQQ	P	14	3	−4	−72.8	−1.6	n.d.b
18	KKSQTSYHHQQMLRTHRV	M	18 (17)	5	0	−72.7	−1.6	n.d.b
	KYDMNFMRDNFWKQQKFK	PC1	0	4	−4	−75.8	−2.1	240
	RFWMEDLTMKIVYWEKFK	PC2	0	7	−6	−73.5	−1.7	0.9

^a^ Position IDs, in the same order: 45, 46, 48, 56, 57, 78, 79, 81, 89, 90, 111, 112, 114, 122, 123, 144, 145 and 147.

^b^ P: permutation; M: mutation; PC: positive control.

^c^ Number of substitutions at 18 variable positions from the corresponding known binder for the P-set designs or from the initial sequence of the common framework-based library for the M-set designs. Number of substitutions from the closest known binder is also shown in parenthesis for the M-set designs.

^d^ Number of poses predicted to bind at the target epitope.

^e^ Net charge.

^f^ Consensus docking score obtained from an arithmetic average of the docked poses at target epitope.

^g^ Calculated from scores over the set of 293 “consensus designs” (see Results section).

^h^ Determined by SPR measurements (see Methods section); weak: K_D_ > 1 μM; n.d.b.: no detected binding.

The two positive-control interfaces, having the 18 variable positions imported from the two known DARPin binders of BCL-W and grafted onto the common framework sequence, were also docked in the same manner. These positive controls had consensus scores of −75.8 and −73.5, corresponding to Z-scores of −2.1 and −1.7, respectively. With the assumption that none of the designs are true binders, the separation of the positive controls from the designs had an AUC of 0.971 (Figure 4). The AUC dropped to 0.922 when best scores were used instead of consensus scores for designs with N_pose_ > 1. While working with rigid scaffolds, this observation suggested the need for structural ensembles to achieve better enrichments and thus motivated the use of consensus scores over best scores in the sections that follow. These positive controls were ranked within the range of the top-18 novel designs, and they were also subjected to experimental testing. It is important to note that to properly compare the scores for the two parental known binders of BCL-W with those of the mutants, we needed to base it on the modeled structures of the known binders rather than their crystal structures. Using the crystal structures would be a case of cognate backbone docking and perfect match in shape complementarity leading to out-of-range scores (DARPin/BCL-W docking scores of −144.5 and −123.3 were obtained for 4k5a [B]/4k5a [A] and 4k5b [B]/4k5b [C], respectively). The overlay of all locus docked poses for the two grafted positive control interfaces is shown in Figure 5B to have the same orientation with those of the selected designs (Figure 5A). These poses are further similarly oriented with those of the known binders, as exemplified in Figure 5C. A closer examination reveals that despite an excellent pose recovery for this cross-docking experiment, there are certain noticeable differences in the fine atomic details at the interface, which are likely due mainly to non-cognate backbone coordinates and to a lesser extent to changes of the framework sequence outside the 18 variable positions. Overall, cross-docking of positive controls predicted that they would retain similar binding relative to the corresponding known binders.

As presented in Table 2, most of these top consensus designs (13 of 18) were from the permutation set despite its smaller representation in the initial library. Also, 15 out of the 18 consensus designs had a net charge equal to that of a positive control (−6 or −4), despite the random sequence generation procedure employed. On average, the top-18 designs were 15 mutations away from the positive-control interfaces, with the closest design being 10 mutations away. These top consensus designs had between 2 and 7 top-1 poses bound at the target epitope. Interestingly, a strong bias towards an increased consensus was observed with 14 out of the 18 novel designs (78%) with at least 3 representative poses bound at the targeted epitope. This level has to be contrasted to only 24% of all consensus designs reaching an N_pose_ > 2. Hence, not only were the top designs predicted to bind stronger to the target, they also did so with a higher number of predicted consensus poses, with an average N_pose_ of 4.2. Comparably, the two positive controls had N_pose_ values of 4 and 7 (Figure 4).

### 3.2 Experimental testing of DARPin designs

**FIGURE 6:**
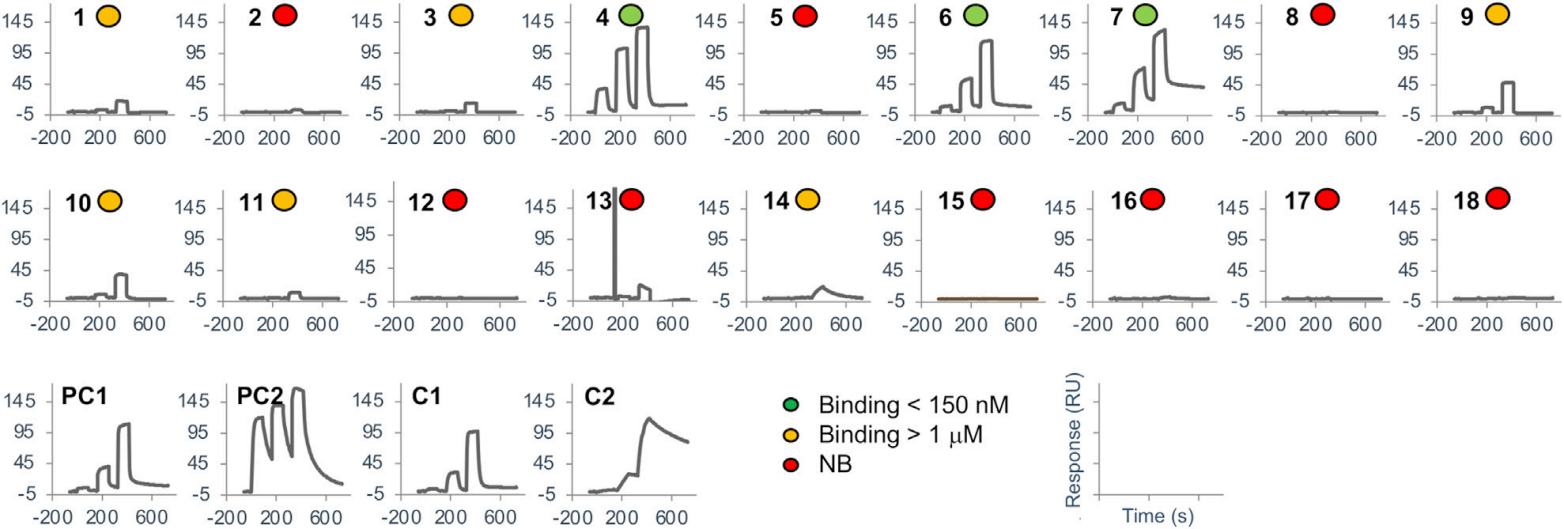
Surface plasmon resonance screening. SPR binding sensorgrams are shown for the 18 top-ranked designs, the positive controls and the known binders. Ranking of designs is based on the consensus score (see also Table 2). Sensorgrams are labeled according to 3 levels of binding affinity as shown in the legend.

The 18 top-ranked consensus designs, together with the 2 positive controls and the 2 parental known binder DARPins were produced in bacteria, purified by IMAC and screened for binding to BCL-W by SPR. The purity levels of the DARPins ranged from 45% to 99% with an average of 82% (Supplementary Table S1). While some of these levels could be considered as suboptimal for SPR experiments and might lead to non-specific binding, they were deemed sufficient for a first-pass screening. Tested DARPins were flowed at a fixed concentration over biotinylated target protein immobilized on the sensorchip. An overview of the SPR binding screen is given in Figure 6. Overall, binding in the nM range was detected for 3 designs, the 2 positive controls and the 2 known binders (Table 2). Additionally, 6 designs had weak binding in the μM range, with a caveat that some of the binding events detected in these cases could be non-specific. Among the top 10 designs, only 3 had no detected binding, while the 3 stronger binders and 4 of the weak binders were present in this group. All 7 binders in the top-10 group belonged to the permutation (P) set. In the group consisting of the 8 remaining tested designs, ranks 11–18, there were only 2 weak binders while the rest of designs had no detected binding. These 2 weak binders were both from the mutation (M) set. Overall, data in Table 2 indicate a certain level of enrichment in binding that follows the predicted docking scores within the set of 18 tested variants, with the caveat that SPR data is insufficient to confirm the predicted binding modes. We also measured the thermal stabilities of the designed DARPins and obtained very high thermostabilities, with melting temperature (T_m_) values typically in the 80–100°C range (Supplementary Table S1), comparable with those measured here for the positive controls and known binders, as well as previously for other DARPins (Schilling et al., 2014a). This stability data provides some level of confidence that the sequence perturbations introduced in the designed variants were able to maintain the folded structure of the archetypical DARPin scaffold.

For the two known DARPin binders of BCL-W, 4k5a [B] and 4k5b [B], we obtained dissociation constants, K_D_, of 26 nM and 3.5 nM, respectively, which are in line with their previously published K_D_ data of 10 nM and 0.64 nM (Schilling et al., 2014a). The two corresponding positive control DARPins, which import only the 18 variable positions of the common framework scaffold from these known binders, bound with K_D_ values of 245 nM and 0.9 nM. These values represent comparable affinities to their respective parental known binders, although it seems that the framework change from the known binders to the common framework sequence impacted detrimentally the 4k5a [B] interface and beneficially the 4k5b [B] interface.

For the 3 novel DARPin designs exhibiting good binding, we obtained dissociation constants, K_D_, in the 40–150 nM range, which are well within the range bracketed by the two positive controls (0.9–245 nM). These were ranked 4, 6 and 7 among the top-18 consensus designs (Table 2), with the 4^th^ ranked design exhibiting the better K_D_ of 44 nM, which is similar to the affinity of one of the known binders (26 nM). Low binding, with K_D_ above 1 μM, could also be detected for designs with ranks 1, 3, 9, 10, 11 and 14. Further details about the binders on their amino-acid substitutions, net charges, sets and substitutions are listed in Table 2.

A retrospective analysis of ranking by best scores instead of consensus scores versus experiment indicated that this approach could also be suitable (Supplementary Table S2). By this ranking of the 18 tested designs, the top 4 gave binding signals and among them the 2^nd^ and 4^th^ ranked are the best designs with K_D_ values of 44 nM and 111 nM. Also, best scoring was able to correctly rank the two positive controls among themselves, i.e., the stronger binder has a more negative score. However, best scoring always performed slightly worse than consensus ranking for the discrimination of binders against non-binders (Supplementary Figure S4).

While our computational strategy forced the designs to bind at a specified locus, our geometric criteria were loose enough to allow for some structural variability around the targeted epitope, that could lead to substantially different structural determinants required for binding. Despite the weak statistics due to the relatively low number of experimentally-validated designs, a close inspection of important structural determinants revealed that the non-binders bury more surface area on average than the validated strong binders (Supplementary Figure S5). Notably, a larger fraction in non-polar surface area on the BCL-W interface is predicted to be lost by the non-binders relative to binders (Supplementary Figure S5). This is an interesting finding to explore in future screening campaigns as docking algorithms are normally calibrated to attribute larger scores to burial of larger interfaces and would indirectly favor or enrich those designs achieving increased surface burial. For this set of binders, hydrophobic residues tend to be preferentially enriched only in the internal DARPin repeat 1 (Supplementary Figure S5).

## 4 Discussion

In this proof-of-concept study, we aimed at exploring if rigid-backbone docking can lead to meaningful biologics discovery. A first objective was to test, in a real-life scenario, the utility of our exhaustive protein-protein docking tool ProPOSE that incorporates side-chain flexibility (Hogues et al., 2018). ProPOSE performed very well in cognate-backbone docking, but returned a lower performance in unbound-backbone docking, thus hampering *de novo* antibody discovery efforts, mainly due to the hypervariable nature of the CDR-H3 loop. While work addressing the challenging problem of backbone sampling and scoring is highly relevant and remains to be pursued, here we explored the practical utility of ProPOSE in its current state by employing a more rigid scaffold, DARPin, which has already been used as an alternative scaffold in biologics discovery (Binz et al., 2003; Pluckthun, 2015). The overarching assumption is that ProPOSE can tolerate some minor level of backbone movements at the binding interface, but the extent of tolerated backbone movements has not been established yet.

From the technological perspective of rigid docking with unbound backbone conformation, employing four experimentally determined backbone conformations, each slightly different from bound backbone conformations, provided a test of the impact of backbone flexibility on biologics design. An initial measure of success was gleaned from so-called positive controls, in which 18 interfacial residues of known DARPin binders to a given target (BCL-W in this study) were transferred to a common DARPin framework sequence, assigned unbound backbone conformations, and cross-docked to the target. The predicted binding modes of these positive controls were similar to those of known binders, but docking scores were reduced almost in half relative to those obtained for the known binders in their bound backbone conformations. Yet, experimental testing of these positive controls showed retained binding affinities at comparable levels relative to the known binders, despite reduced scores. This established a new range of binding scores at a reduced magnitude which was adapted for cross-docking but remained predictive of true-positive binders. Consequently, novel DARPin designs cross-docked at that same target epitope were top-ranked and had scores within the re-established score level suitable for cross-docking. Upon their experimental testing, seven out of top-10 ranked designs demonstrated at least some level of binding to the target, with 3 of them exhibiting binding strengths similar to those of the positive controls as well as the previously known binders.

Despite this initial relative success, rigid-backbone docking remains challenging even for scaffolds with fairly rigid protein backbone like DARPins. Several limitations of this approach and directions for possible improvements are noted below.

First, most novel binders belonged to the random permutation (P) set, which confines the library space with respect to certain global properties, for example, the net charge. These results thus point to the benefits of landing into the “right” regions of the library space after randomization at variable positions. While it was certainly harder for members from the random mutation (M) set to reach the top of the hit list, the finding of two weak binders belonging the M-set is extremely encouraging. In principle, real-life applications utilize mainly M-libraries. One way in this direction could be to enlarge the size of the docked diversified sub-library (only ∼2,000 in this study). This could be feasible with access to large computing resources given the not overly prohibitive computational task involved in running ProPOSE. An alternative approach could be a focused expansion into P-subsets around initial M-set hits from a relatively sparse sub-library. This approach could set a preferred range for net charge, for example, and it would be especially beneficial as the number of randomized interfacial positions increases. Furthermore, the efficiency of the M-libraries at finding better hits could most likely be improved by applying structure-guided filters to search in more relevant regions of the sequence space. For instance, designs could be filtered based on their complementarity in charge or by their exposure of polar or non-polar surfaces at variable positions based on structural information of the selected binding epitope being targeted.

Secondly, while initial hits are often weak binders which are difficult to characterize, they should not be immediately discarded but rather treated as seeds for further optimization by affinity maturation, which can be done either experimentally (e.g., display methods) or computationally (e.g., ADAPT platform). This aspect has significant practical importance, given that by random sampling of the immense library space it is highly unlikely to obtain a very strong binder.

Thirdly, the unbound backbone conformations selected for cross-docking were from experimentally determined crystal structures. This is similar to the multiple protein structure approach used in small-molecule docking and virtual screening (Sheridan et al., 2008). Because the DARPin scaffold is not completely rigid and scoring functions used in docking are sensitive to atomic positions, including more than one backbone as templates in the cross-docking approach was felt to be beneficial. Carefully derived simulated structures obtained, for example, via backrub motions, molecular dynamics or Monte-Carlo simulations can be used as alternatives sources to experimentally-determined backbone conformations. The multiple template approach used here for docking was also extended to the stage of hit ranking, via consensus scoring. This seemed to provide a reasonable enrichment, although retrospectively we also found that the best-score approach might provide a similarly good, if not better ranking, among the small set of hits ranked by consensus scoring.

Despite some approximations in the underlying methodology adopted here, it is encouraging that cross-docking could identify binding sequences that differ substantially from known binders out of thousands of potential candidates. This relative success may be attributed to the foundational work underlying the methods used here to address the two intimately-related challenges of docking and scoring in computational drug discovery (Schneider et al., 2022). On one hand, for binding mode prediction, ProPOSE was used given its high accuracy in rigid-backbone docking when the bound-backbone conformation is provided. On the other hand, for ranking among different docked variants, ProPOSE employed a scoring function drawn from the solvated interaction energy (SIE) exhibiting high transferability from small-molecule to protein ligands (Purisima et al., 2023).

The data presented here support the notion that *de novo* biologics discovery *via* computational methods is a tractable problem that could complement the more traditional and matured wet-lab methods of library display screening and animal immunization. One main added benefit of the structure-based approach is directing the binding response towards desired target locations, e.g., functionally relevant, in a controlled manner. Further advances in several areas such as backbone sampling and depth of theoretical library screening, will be required for maturing *de novo* biologics discovery for routine applications in the not-so-distant future.

## Data Availability

The original contributions presented in the study are included in the article/Supplementary Material, further inquiries can be directed to the corresponding author.
