# Supplementary material for: Exploring rigid-backbone protein docking in biologics discovery: a test using the DARPin scaffold
Source: Front Mol Biosci. 2023 Aug 24;10:1253689. doi: 10.3389/fmolb.2023.1253689 (PMC10484509; doi:10.3389/fmolb.2023.1253689)
Supplement: Supplementary file 1 [file DataSheet1.PDF]

Supplementary Material

Exploring Rigid-Backbone Protein Docking in Biologics Discovery: A Test Using the DARPin Scaffold

Francis Gaudreault[1], Jason Baardsnes[1], Yuliya Martynova[1], Aurore Dachon[1], Hervé Hogues[1], Christopher R. Corbeil[1], Enrico O. Purisima[1], Mélanie Arbour[1], Traian Sulea[1,2*]

[1]Human Health Therapeutics Research Centre, National Research Council Canada, Montreal, QC, H4P 2R2, Canada

[2]Institute of Parasitology, McGill University, Sainte-Anne-de-Bellevue, QC, H9X 3V9, Canada

*** Correspondence:**
Traian Sulea
traian.sulea@nrc-cnrc.gc.ca

1 Supplementary Data

1.1 Final BCL-W sequence: contains an Avitag (underlined), a bacteriophage lambda protein D fusion tag (italic), the amino acids 2-171 of BCL-W Uniprot Q92843 (bold) and a poly-His tag. BCL-W protein sequence is the same as in (Schilling et al., 2014), including the mutations P117V and Q133R.

MAGLNDIFEAQKIEWHEGS*MGTATAPGGLSAKAPAMTPLMLDTSSRKLVAWDGTTDGAAVGILAVAA*
*DQTSTTLTFYKSGTFRYEDVLWPEAASDETKKRTAFAGTAISIV*GS**ATPASAPDTRALVADFVGYKL**
RQKGYVCGAGPGEGPAADPLHQAMRAAGDEFETRFRRTFSDLAAQLHVTPGSAQQRFTQVSDELFQG
GPNWGRLVAFFVFGAALCAESVNKEMEVLVGQVQEWMVAYLETRLADWIHSSGGWAEFTALYGDGAL
EEARRLREGNWASVREASHHHHHH

1.2 SDS-PAGE gels of produced DARPins

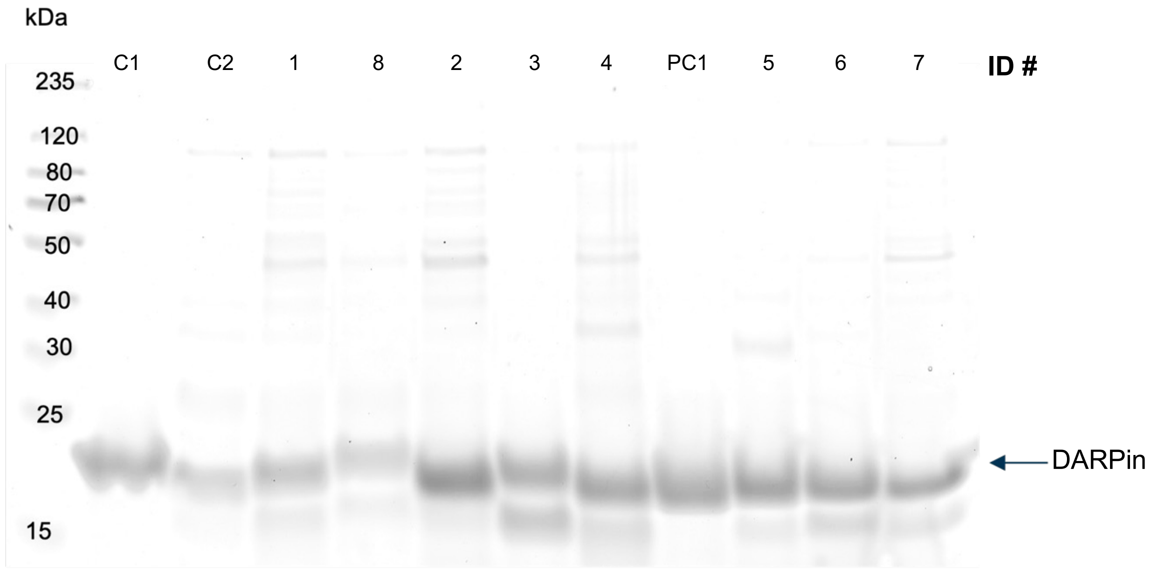

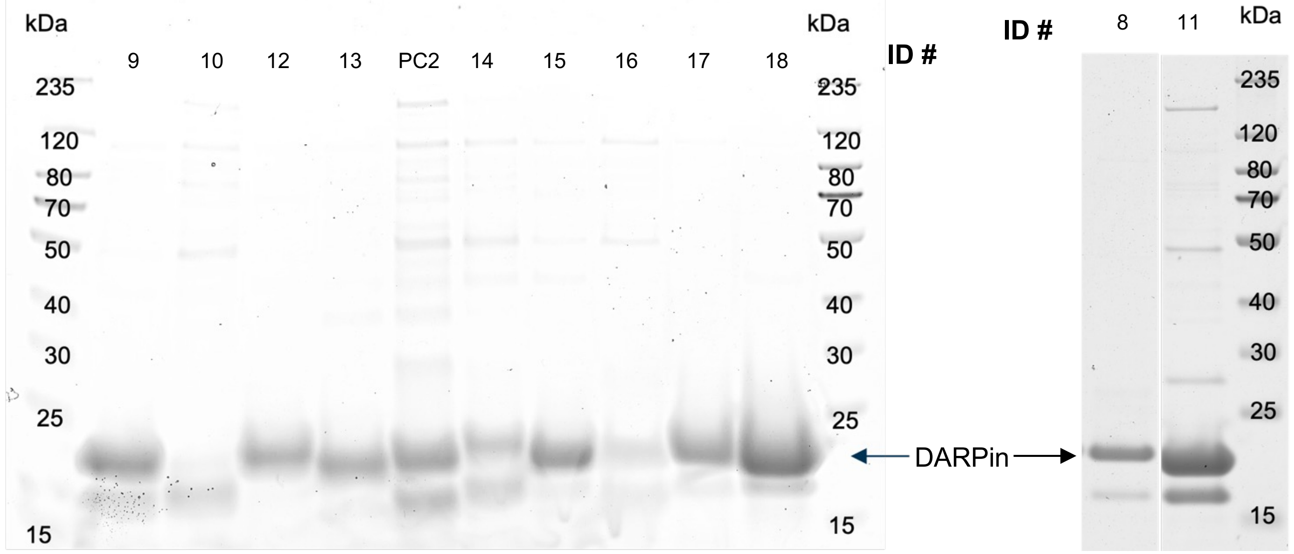

1.3 DSC thermograms of produced DARPins

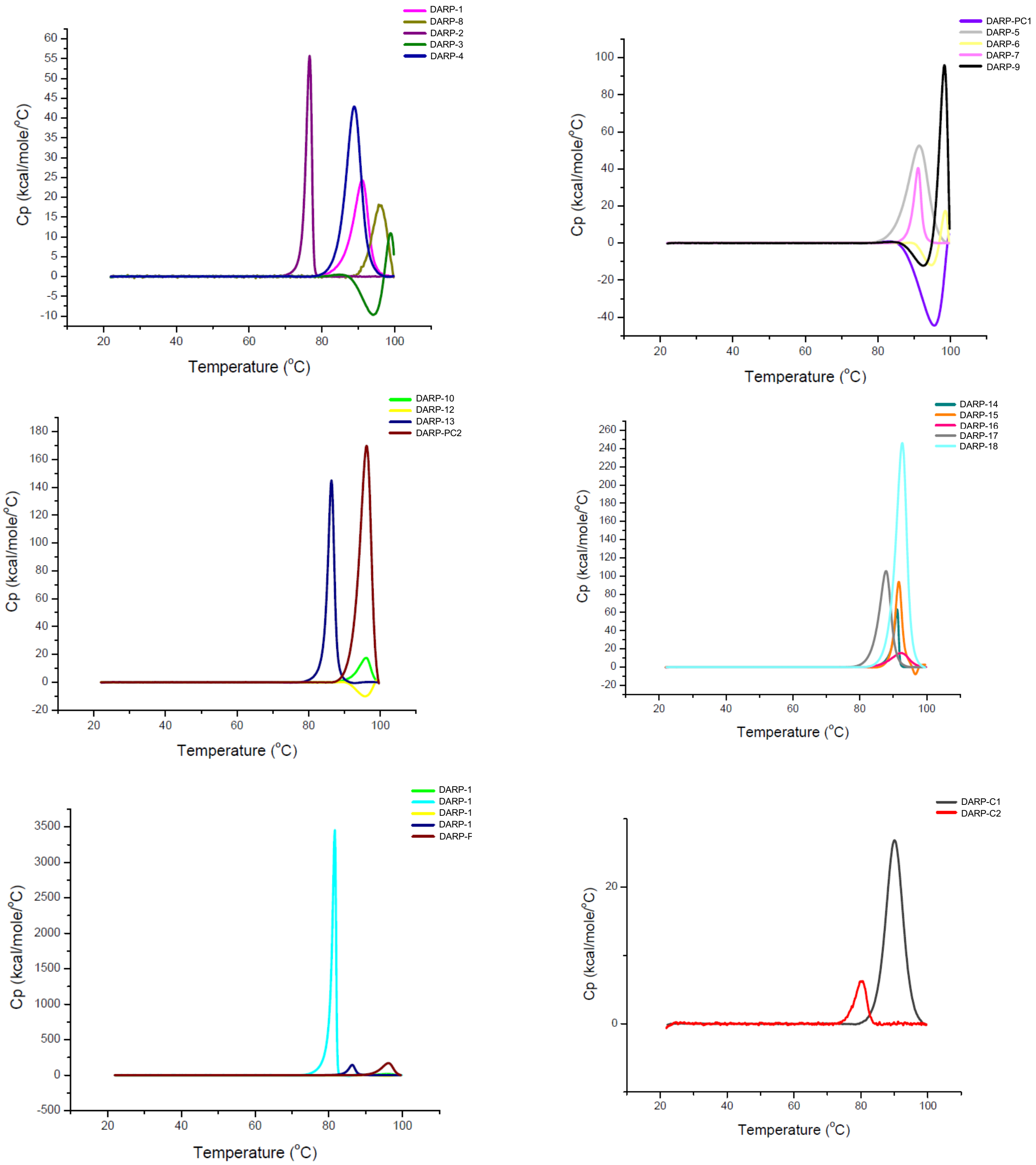

2 Supplementary Tables and Figures

Table S1. Biophysical characterization of the purified proteins under study.

Protein[a]	MW (kDa)	pI	Extension Coefficient	Purity[b] (%)	T_m[c] (°C)	Concentration (μg/mL)	Molarity (μM)	Volume (mL)
**DARPin-1**	18.2	5.28	12490	80.8	91.2	354	19.4	2.5
**DARPin-2**	18.3	5.78	12490	82.8	76.7	369	20.2	2.5
**DARPin-3**	18.2	5.28	12490	89.3	> 100.0	400	21.9	2.5
**DARPin-4**	18.2	5.28	12490	77.8	89.0	347	19.0	2.5
**DARPin-5**	18.2	5.28	12490	91.1	91.3	405	22.2	2.5
**DARPin-6**	18.3	5.54	6990	78.9	> 100.0	352	19.3	2.8
**DARPin-7**	18.2	5.28	12490	86.0	91.0	385	21.1	2.5
**DARPin-8**	18.3	5.54	6990	88.7	95.6	155	8.5	2.5
**DARPin-9**	18.3	5.54	6990	75.5	98.3	323	17.7	2.5
**DARPin-10**	18.2	5.28	12490	77.8	96.1	263	14.4	5.0
**DARPin-11**	18.1	5.54	8480	70.5	N/A	456	25.3	2.5
**DARPin-12**	18.3	5.54	6990	98.8	> 100.0	427	23.4	5.0
**DARPin-13**	18.2	5.28	12490	90.8	86.4	309	17.0	2.5
**DARPin-14**	18.4	5.63	22460	49.4	91.2	208	11.3	2.5
**DARPin-15**	18.2	5.54	6990	89.6	91.6	385	21.2	2.5
**DARPin-16**	18.2	5.28	12490	61.2	92.5	186	10.2	2.5
**DARPin-17**	18.3	5.54	6990	94.2	87.8	420	23.0	2.5
**DARPin-18**	18.1	5.87	1490	90.5	92.6	382	21.1	2.5
**DARPin-PC1**	18.3	5.54	6990	92.7	> 100.0	412	22.6	2.5
**DARPin-PC2**	18.2	5.28	12490	44.6	96.1	191	10.5	2.5
**DARPin-C1**	18.1	5.66	6990	97.7	90.0	442	24.4	5.0
**DARPin-C2**	18.1	5.33	17990	92.5	80.5	115	6.3	2.5
**BCL-W**	31.5	5.32	53065	100.0	-	980	31.1	1.0

[a] DARPin #: rank number; PC: positive control; C: known binder.
[b] The purity levels of the final proteins were measured by densitometry from SDS-PAGE image using Image Lab (Bio-Rad Laboratories Inc, Version 6.1.0). The purity corresponds to the percentage of the band's volume compared to the entire volume of the lane. The SDS-PAGE gels of the DARPins were provided as Supplementary Data.
[c] The melting temperatures were estimated using Differential Scanning Calorimetry (DSC). DSC analysis was performed using the Malvern MicroCal DSC system by ramping the temperature from 20 to 100°C with a scan rate of 1°C/minute. Data were analyzed using Origin7 Software with manual baseline assignment. Entries that exceeded the boundaries of the melting curve were reported as T_m > 100°C. DARPin-11 was reported as N/A due to insufficient material. The DSC thermograms were provided as Supplementary Data.

Table S2. Selected consensus designs re-ranked by best score instead of consensus score.

Rank	Variable positions[a]	Set[b]	N_{sub}[c]	N_{pose}[d]	Q_{net}[e]	Score[f]	K_D (nM)[g]
1	RMTKEKFFWEILWYDMVK	P	14	7	-6	-92.2	weak
2	KFWMEMLTDWIYEVRKKF	P	10	3	-6	-87.4	44
3	RAVNRTVFVYWAYNFRVV	M	18(16)	2	-4	-86.8	weak
4	VWWEEDFKIKMMKFYTLR	P	14	3	-6	-85.3	111
5	KYRKNKFWFNDQFKDQMM	P	14	3	-4	-85.0	n.d.b.
6	KFWFETMDKMKRYEWVIL	P	14	7	-6	-85.0	weak
7	FKMWEMLFWRVIYEDKKT	P	14	5	-6	-84.0	n.d.b.
8	RQIVHRHWFDVIKYWRHL	M	18(17)	3	-1	-82.9	n.d.b.
9	KFMREEFWWLIKKTDYMV	P	15	6	-6	-82.7	n.d.b.
10	KIMWFKWDYKELMVETFR	P	15	3	-6	-80.7	weak
11	KFWYNDFQMDFQMRNKKK	P	13	4	-4	-80.6	150
12	KYWYRTTWYHAIWNFYKQ	M	18(16)	5	-3	-79.9	weak
13	KLMEYDFMVWITKFERWK	P	14	7	-6	-79.5	n.d.b.
14	RKMDQKFKMNDYWNFQFK	P	15	2	-4	-79.2	weak
15	KFWMQRFMQYKDFKDKNN	P	15	2	-4	-79.1	n.d.b.
16	KFFRNNKMDYWKKMDFQQ	P	14	3	-4	-78.9	n.d.b.
17	KKSQTSYHHQQMLRTHRV	M	18(17)	5	0	-75.9	n.d.b.
18	KYFEWVQRVMFKVVLMNR	M	18(14)	2	-4	-75.3	n.d.b.
	KYDMNFMRDNFWKQQKFK	PC1	0	4	-4	-78.6	240
	RFWMEDLTMKIVYWEKFK	PC2	0	7	-6	-84.1	0.9

[a] Position IDs in the same order: 45, 46, 48, 56, 57, 78, 79, 81, 89, 90, 111, 112, 114, 122, 123, 144, 145 and 147.
[b] P: permutation; M: mutation; PC: positive control.
[c] Number of substitutions at 18 variable positions from the corresponding known binder for the P-set designs or from the initial sequence of the common framework-based library for the M-set designs. Number of substitutions from the closest known binder is also shown in parenthesis for the M-set designs.
[d] Number of poses predicted to bind at the target epitope.
[e] Net charge.
[f] Best docking score among docked poses at target epitope.
[g] Determined by SPR measurements (see Methods section); weak: $K_D > 1$ µM; n.d.b.: no detected binding.

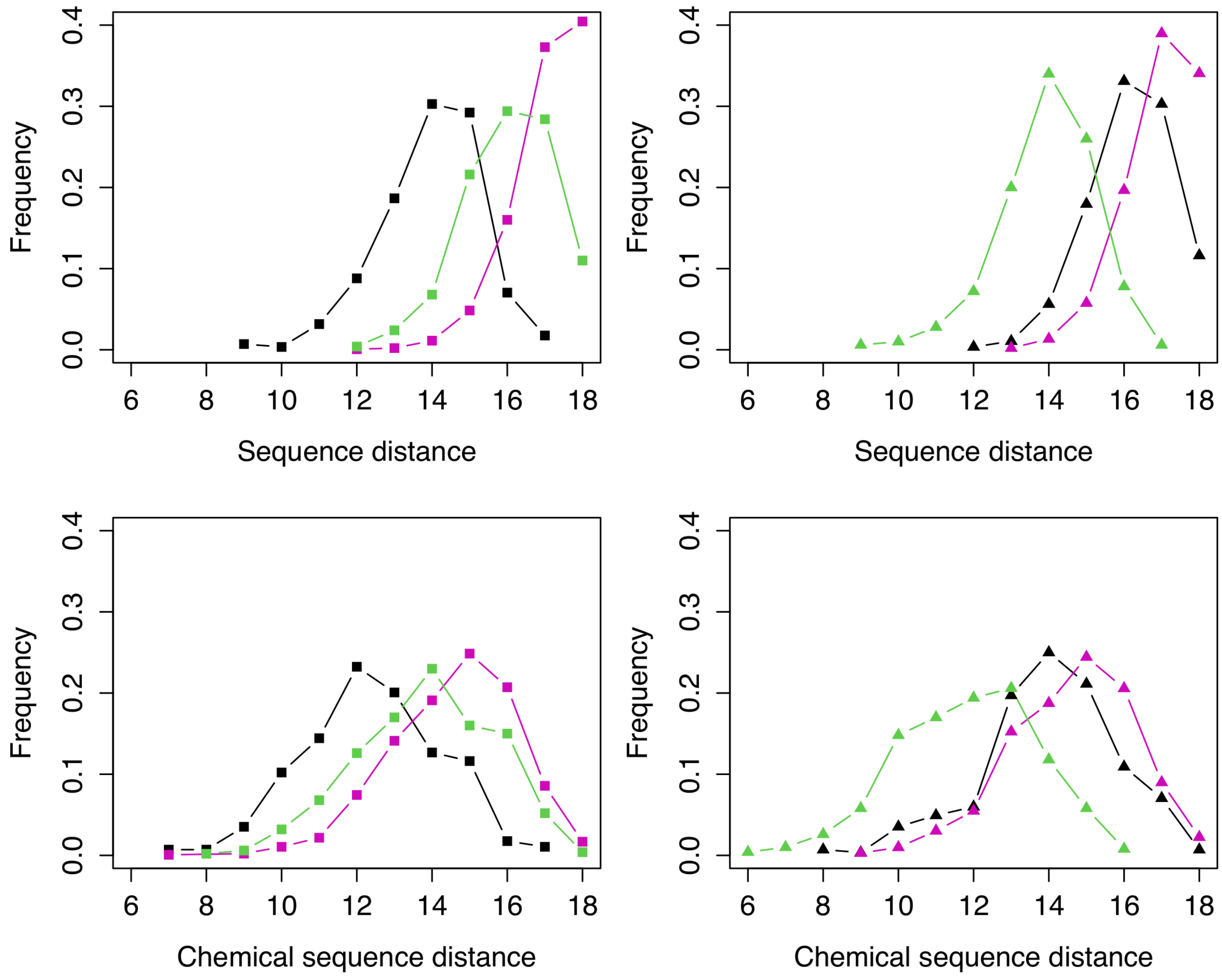

Supplementary Figure S1. Sequence distances of library designs to positive controls. Frequency of library designs as a function of sequence distance within the single-point mutation-based set (black; N=1429) and the permutation-based set from parent 4k5a[B]/PC1 (pink; N=284) and parent 4k5b[B]/PC2 (green; N=500) as a function of the distance and chemical distance. The reference sequence used in the comparison is PC1 (square) and PC2 (triangle). The sequence distance is the number of mutations away from the reference. The chemical sequence distance is the number of alphabet group changes from the reference.

Permutation-based set

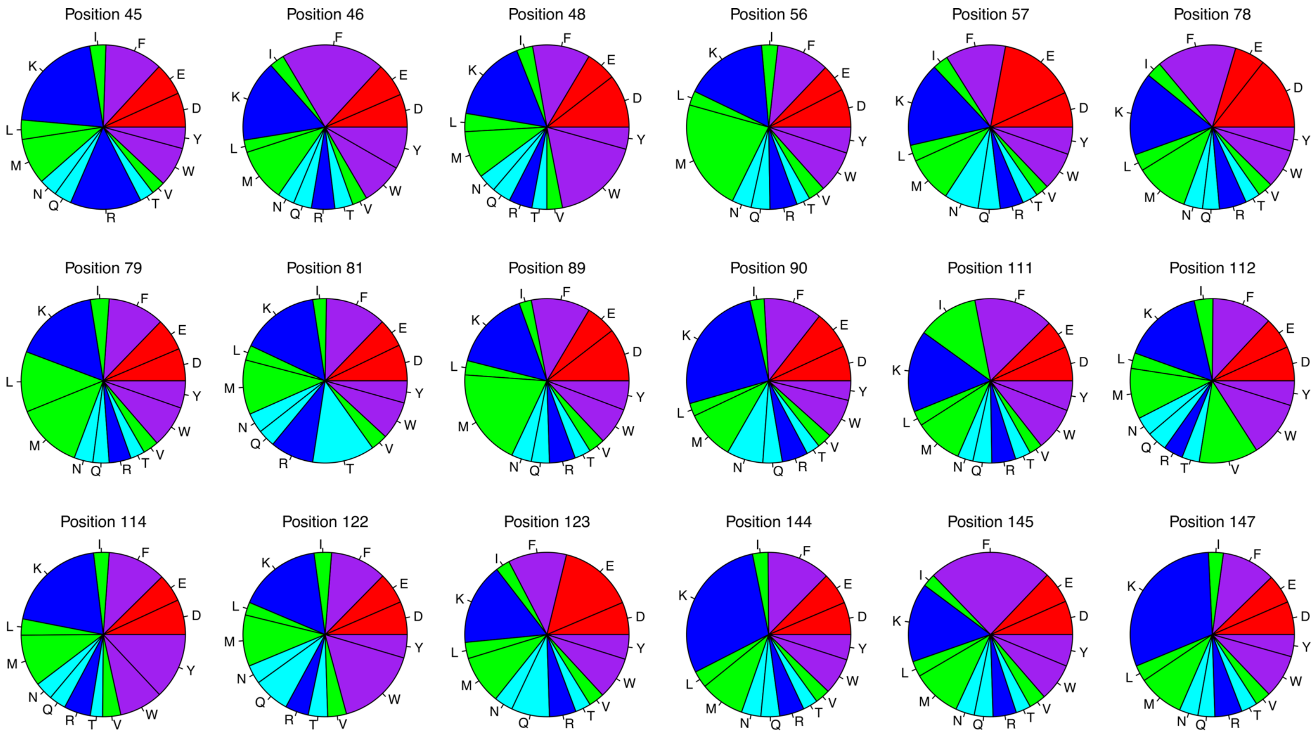

Mutation-based set

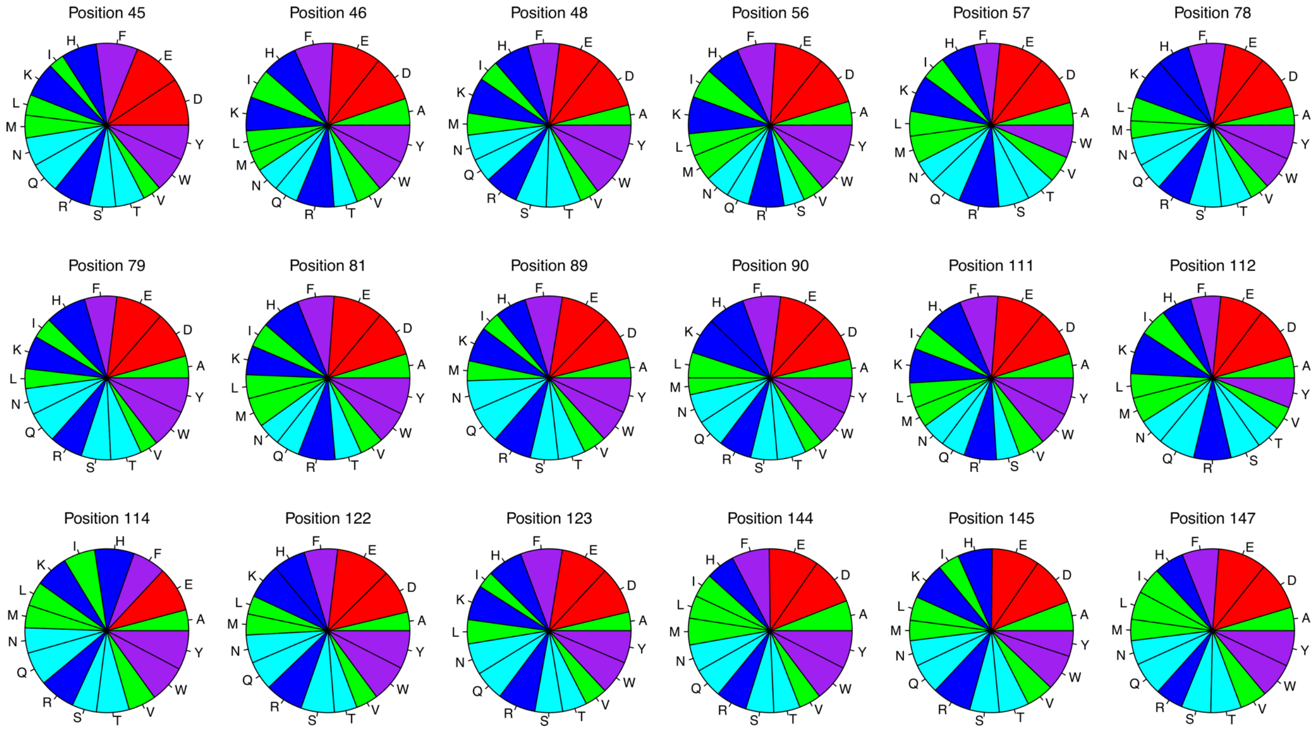

Supplemental Figure S2. Proportions of amino acids in the library. Proportions of amino acids at each variable position for designs part of the permutation-based and mutation-based sets.

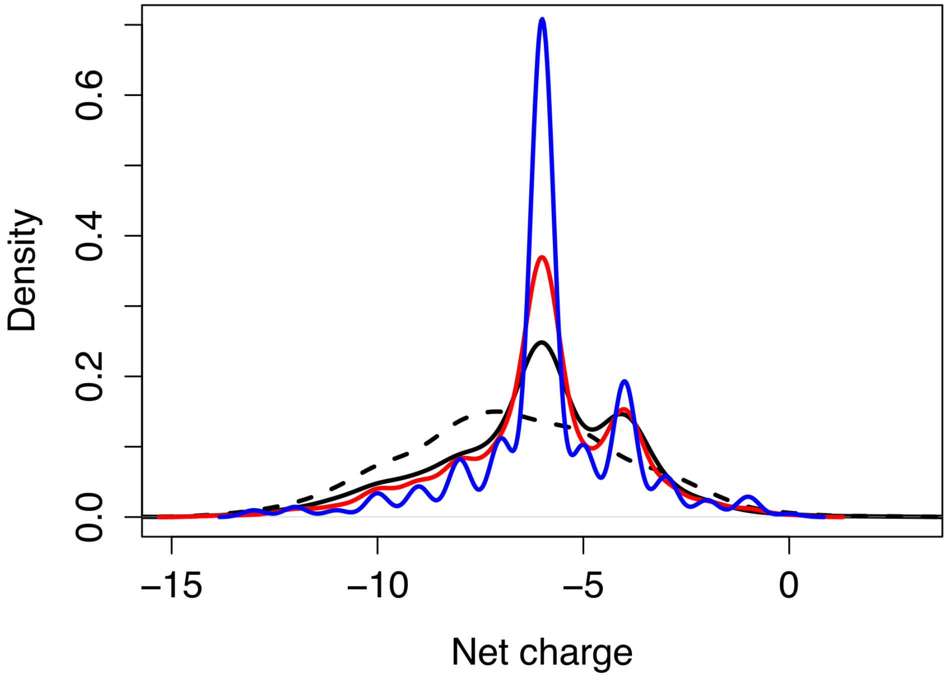

Supplemental Figure S3. Net charge distribution of the library designs. Density plots highlighting the frequency of designs as a function of the net charge for the designs contained in the whole library (black solid line; N=2,213), contained in the mutation-based set (black dashed line; N=1,429), successfully predicted to bind at the epitope of known binders without consensus in pose prediction (red; N=1,033) or with consensus (blue; N=295). The density plots were smoothed using a bandwidth of 0.65. The net charges of the two positive controls are –4 and –6.

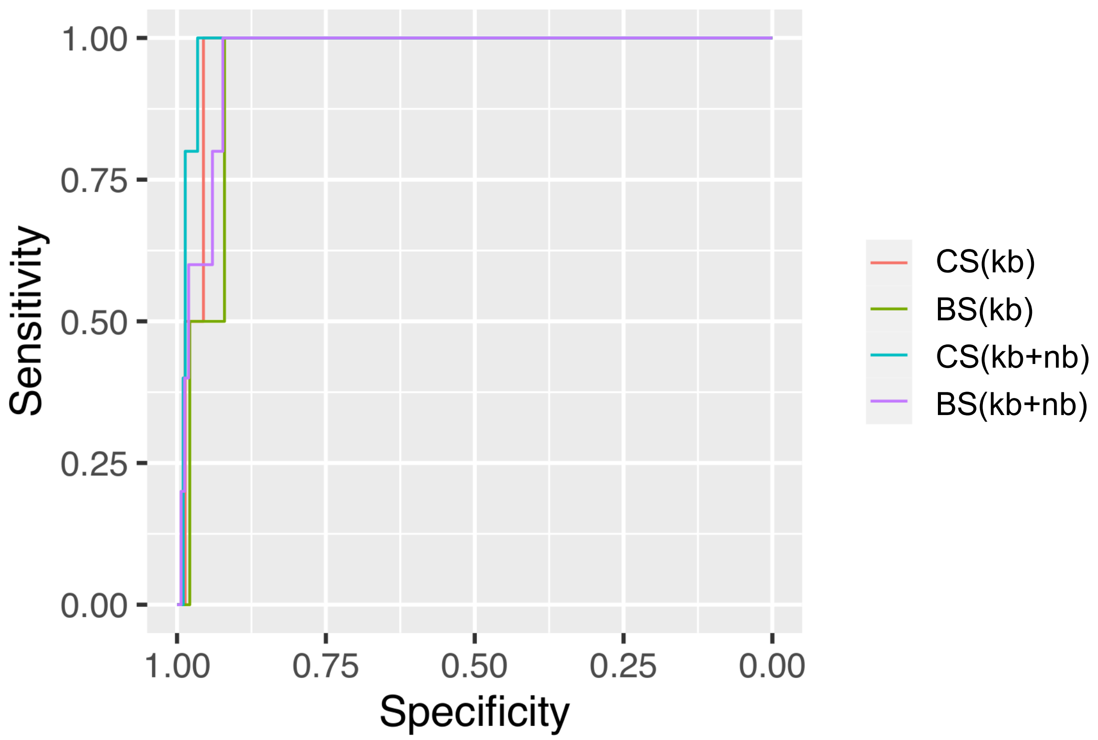

Supplemental Figure S4. Discrimination of binders against non-binders. Receiver operating characteristic (ROC) curves are plotted when the locus designs were clustered then ranked by consensus scores (CS) or ranked by best docking scores (BS). The set of true positives included either the two known binders (kb) or the two known binders and three novel binders from this study (kb+nb). The corresponding areas under curve (AUC) values were 0.971, 0.950, 0.983 and 0.965 for CS(kb), BS(kb), CS(kb+nb) and BS(kb+nb), respectively.

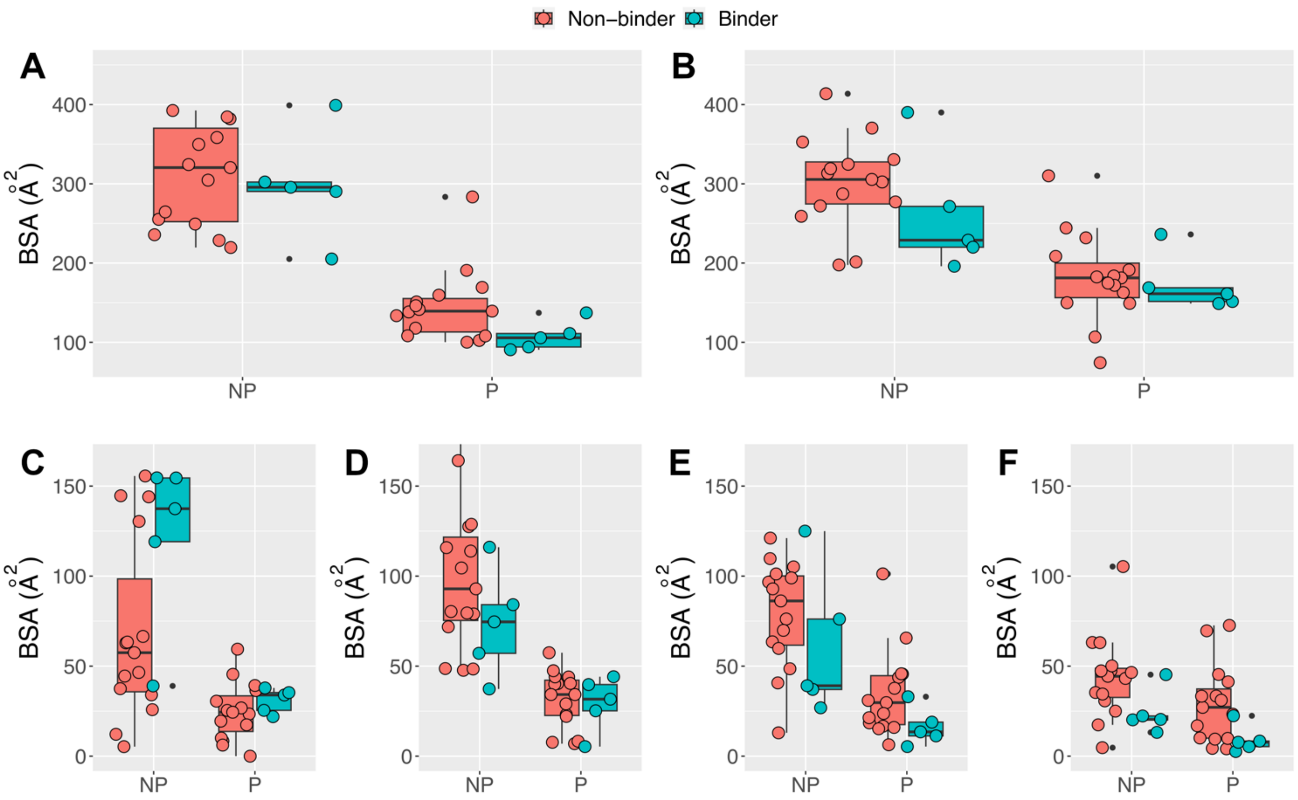

Supplemental Figure S5. Surface area distribution for the experimentally-validated designs. Distribution in buried surface area (BSA) for the set of non-binders (weak or no detected binding according to **Table 2** in the main paper) and for the set of binders (two positive controls and three designs with $K_D \leq 1$ μM). The BSA values were calculated for the set of polar (P) and non-polar (NP) atoms as part of (**A**) the entire DARPin molecule, (**B**) the BCL-W interface, and (**C** to **F**) for internal repeats 1 to 4, respectively, of the DARPin molecule. Only oxygen and nitrogen atoms were classified as polar. Only the pose that scored best within a cluster of the consensus design was used in the analysis.

References

Schilling, J., Schoppe, J., and Pluckthun, A. (2014). From DARPins to LoopDARPins: novel LoopDARPin design allows the selection of low picomolar binders in a single round of ribosome display. *J Mol Biol* 426(3)**,** 691-721. doi: 10.1016/j.jmb.2013.10.026.
